# Clinical characteristics and distinctiveness of DSM-5 eating disorder diagnoses: findings from a large naturalistic clinical database

**DOI:** 10.1186/2050-2974-1-31

**Published:** 2013-08-20

**Authors:** Kerstin Ekeroth, David Clinton, Claes Norring, Andreas Birgegård

**Affiliations:** 1 Resource Center for Eating Disorders/Center for Psychiatry Research Stockholm, Department of Clinical Neuroscience, Karolinska Institutet, Stockholm, Sweden; 2 Stockholm Centre for Eating Disorders, Stockholm County Council, Stockholm, Sweden

**Keywords:** Eating disorders, DSM-5, Clinical distinctiveness, Purging disorder

## Abstract

**Background:**

DSM-IV eating disorder (ED) diagnoses have been criticized for lack of clinical utility, diagnostic instability, and over-inclusiveness of the residual category “ED not otherwise specified” (EDNOS). Revisions made in DSM-5 attempt to generate a more scientifically valid and clinically relevant system of ED classification. The aim with the present study was to examine clinical characteristics and distinctiveness of the new DSM-5 ED diagnoses, especially concerning purging disorder (PD).

**Methods:**

Using a large naturalistic Swedish ED database, 2233 adult women were diagnosed using DSM-5. Initial and 1-year follow-up psychopathology data were analyzed. Measures included the Eating Disorder Examination Questionnaire, Structural Eating Disorder Interview, Clinical Impairment Assessment, Structural Analysis of Social Behavior, Comprehensive Psychiatric Rating Scale, and Structured Clinical Interview for DSM-IV Axis I Disorders.

**Results:**

Few meaningful differences emerged between anorexia nervosa binge/purge subtype (ANB/P), PD, and bulimia nervosa (BN). Unspecified Feeding and Eating Disorders (UFED) showed significantly less severity compared to other groups.

**Conclusions:**

PD does not appear to constitute a distinct diagnosis, the distinction between atypical AN and PD requires clarification, and minimum inclusion criteria for UFED are needed. Further sub-classification is unlikely to improve clinical utility. Instead, better delineation of commonalities is important.

## Background

Compared to its predecessor DSM-5 [1] attempts to generate a more scientifically robust and clinically relevant system of classification for eating disorders (ED). Although DSM-IV was fraught with problems and changes were necessary, the consequences of the revisions remain unclear. Since DSM-5 will have considerable impact on the future of both scientific and clinical work, research on the clinical characteristics and distinctiveness of the now finalized changes will inform about the utility and consequences of these amendments. DSM-IV has been criticized for lack of clinical utility, diagnostic instability, general irrelevance, and the over-inclusiveness of the residual category of “ED not otherwise specified” (EDNOS), a group that may encompass 50 to 70% of patients in tertiary care and outpatient settings [2,3], but which has been the subject of little research. In order to better define and limit EDNOS, DSM-5 lowers thresholds for AN and BN, and establishes binge eating disorder (BED) as a separate diagnosis. These changes are supported by research [4-6] and will likely decrease EDNOS to some extent [7-9]. However, many patients will still be classified under a “residual” or unspecified diagnostic category. Temporal instability is another major problem for DSM-IV [10-12], with many patients migrating to a diagnosis other than their initial one over time [13]. What’s more, knowledge about the stability of EDNOS is limited [14], and many patients develop other EDs [12,15]. While some studies suggest that EDNOS represents a transitional stage of progression into or out of full ED, other work suggests that these patients constitute a mixed group, as seriously ill as AN or BN [2].

DSM-5 [1] renames EDNOS as Other Specified Feeding and Eating Disorders (OSFED) and includes sub-categories for atypical AN (AAN), subthreshold BN (SubBN), purging disorder (PD), and night eating syndrome (NES). Although potentially a step forward, these OSFED “subtypes” are problematic. AAN includes the unspecified criterion “significant weight loss” (in normal weight or overweight individuals), which makes reliable research and clinical evaluation difficult. Also, since SubBN seems to be uncommon in clinical samples, its utility may be limited [7]. Additionally, DSM-5 includes a totally undefined category called Unspecified Feeding or Eating Disorder (UFED).

Perhaps the most researched of the new OSFED subcategories is PD. However, empirical support for PD is equivocal at best and studies have often found contradictory or no differences between PD and other EDs [16-20]. PD does not appear to differ in remission rates compared to other diagnoses [20-22], and studies of the persistence of symptoms present a varied picture [21-23]. Most work has compared PD to BN, and a few to AN and BED, but no comparisons have been made to other EDNOS categories, which is imperative if PD is to be considered meaningfully different from these, and expected to emerge in the future as a distinct diagnosis of its own [24].

Research on clinical characteristics, co-morbidity, outcome and diagnostic stability from large samples is needed to inform on the relevance and validity of the DSM-5. The aim of the present study was to examine the clinical characteristics and distinctiveness of DSM-5 ED diagnoses in general and PD specifically. It concerned ED-related and general psychopathology, co-morbidity, and outcome^a^.

## Methods

### Participants

Data was drawn from the Stepwise quality assurance database, a large-scale internet-based data collection system for specialized ED care in Sweden. The database has been in use since 2005, and data for the present study came from 34 treatment units. Inclusion criteria were medical or self-referral to one of the participating treatment units, a diagnosed DSM-IV ED, plus intention to treat the patient at the unit in question. Baseline data from 2233 adult women registered after February 2008 (when a change in methodology was made), were available for the present study. Age ranged between 18 and 67 years (*M* = 25.7; *S*.*D*. = 7.84). DSM-IV ED diagnoses were: 16.4% AN (61.9% restrictive subtype, RAN), 31.7% BN (70.4% purging subtype), and 51.9% EDNOS (20.4% subthreshold AN, 14.2% subthreshold BN, 30.5% purging/compensatory behavior, 10.4% binge eating, and 24.5% other symptoms or variants). Of the complete sample 937 (42%) patients had not yet reached the 12-month follow-up point at the time of data extraction, thus only 1296 participants were considered eligible. From this group, 55% (530) had complete 12-month data and were considered representative of the complete sample after several comparative analyses were conducted. The first analysis compared patients with and without follow-up data on the initial assessments, and found no other differences besides those with follow-up data scoring somewhat higher on SASB ‘self-emancipation’ and ‘self-control’ than those without follow-up data^b^. The second analysis compared mean differences between the complete sample and the group with follow-up data within each respective diagnosis and no or only small effects (*d* < 0.27; *p* < 0.01) were found. Finally, we conducted the main analyses separately for the group of patients with follow-up data (N = 530) and results did not differ significantly compared to analyses on the entire sample (if anything, differences were fewer and smaller, and thereby in the direction of our conclusions).

In this clinical, naturalistic database, attrition was due to the following: patients moving to another treatment unit (15% of those without follow-up data), not having been eligible for treatment (for example due to having no full-syndrome ED; 31%), being abroad or otherwise indisposed during the eight-week time window for follow-up (33%), or having declined further follow-up (21%).

### Procedure

Patients were assessed by ED specialists prior to treatment, usually within the first three visits. Individuals in need of inpatient care generally completed assessment during the first week of treatment. Assessment took about 80 minutes and included demographic information, psychiatric history, clinical ratings and interviews, as well as questionnaires. All patients approved using their data for research purposes.

### Measures

#### Eating Disorder Examination Questionnaire (EDE-Q)

A 36-item measure of general ED symptoms and features, comprising four sub-scales of Restraint, Eating Concern, Shape Concern, and Weight Concern, plus a global score [25]. The EDE-Q has shown good psychometric properties [26]. Global score was used as a measure of overall eating psychopathology, while single items were used for operationalization of DSM-5 diagnoses (see below). In the present study, Cronbach’s alphas were 0.82 for Restraint, 0.70 for Eating Concern, 0.89 for Shape Concern, and 0.74 for the Weight Concern scale.

#### Structured Eating Disorder Interview (SEDI)

A semi-structured clinical interview developed specifically for Stepwise. It is based directly on the DSM-IV ED criteria and consists of 20–30 questions, depending on what follow-up questions need to be asked. SEDI was used for operationalization of ED-diagnoses. Preliminary validation against the EDE-interview has shown a good concordance of 81% concerning specific ED diagnoses (including EDNOS and BED) and Kendall’s Tau-b of τ = 0.69 (*p* < 0.001)[27].

#### Structured Clinical Interview for DSM-IV Axis I Disorders (SCID-I)

A semi-structured diagnostic interview for assessing DSM-IV Axis I disorders. The present study used data on mood, anxiety, and substance abuse disorders [28]. SCID-I has shown fair to good psychometric properties [29-31].

#### Comprehensive Psychiatric Rating Scale - Self-rating Scale for Affective Syndromes (CPRS-S-A)

Consisting of 19 items measuring anxiety, depression and compulsiveness [32] which are common problems related to ED [33,34]. Because of the high intercorrelation between the scales (*r* = 0.78 - 0.86), a total/global symptom index ranging from 0 (no distress) to 3 (high distress) was used. In the present sample, Cronbach’s alphas were 0.88, 0.81, and 0.85 for the Depression, Anxiety and Compulsiveness scales respectively. CPRS-S-A has shown good psychometric properties [32,35].

#### Clinical Impairment Assessment (CIA; version 3.0)

Is a 16-item questionnaire assessing the severity of psychosocial impairment caused by ED behaviors and attitudes covering mood, self-perception, cognitive functioning, interpersonal functioning, and work performance during the last 28 days [36,37]. Cronbach’s alpha for the present sample was 0.92. The CIA has shown good psychometric properties in clinical [36,37], high risk [38] and community samples [39].

#### Structural Analysis of Social Behavior (SASB), intrex questionnaire

A 36-item questionnaire based on two orthogonal dimensions (affiliation and interdependence) that measures eight aspects of self-image [40]. The present study used the SASB variables of Self-emancipation (letting oneself go, not having strong principles) and Self-control (monitoring and restraining self), along with the Affiliation vector (i.e. the balance between positive and negative self-image, calculated by weighting and combining six affiliation-related scale scores), which corresponds to a measure of general self-esteem. SASB has previously been found to have relevance with respect to EDs [41,42], and has shown good psychometric properties [40,43,44]. Cronbach’s alpha for the clusters Self-emancipation and Self-control were 0.55 and 0.68 respectively, and 0.90 for the Affiliation vector using the relevant scales as items.

### Definition of purging disorder and operationalization of DSM-5 ED

A more detailed description of the operationalization of previously proposed DSM-5 ED diagnoses has been presented previously [7], and details are available upon request. “EDNOS other”, not independently demarcated in the SEDI interview, was defined as at least one psychological symptom (weight phobia, self-esteem unduly affected by weight or body shape, disturbed body experience, or denial of seriousness of low weight), in addition to at least one behavioral symptom (binge eating, loss of control during eating, purging, or fasting/exercise) or physical (anorexic weight or amenorrhea) symptom. Since the study was designed prior to final publication of DSM-5 operationalization of DSM-5 ED diagnoses was based on information from http://www.dsm5.org as of October 2012. Applying SPSS syntax, diagnoses were based on SEDI and EDE-Q variables. Since the SEDI only assesses whether behaviors have been present at least twice a week for at least three months and the EDE-Q only asks about frequency of bingeing and compensation for the last month, it was not possible to be certain about the fulfillment of the DSM-5 criteria specifying frequency and duration of binge eating and compensatory behavior for once a week for at least three months. EDE-Q frequency items (for example concerning OBE) were only used to establish the lower DSM-5 criteria in cases where patients did not already meet DSM-IV criteria. Since DSM-5 criteria for PD lack specification of both frequency and duration the SEDI item assessing “regular purging” was used, which implies that it is not an occasional behavior. To avoid including underweight patients who could otherwise fulfill criteria for AN, only individuals with a BMI over 18.5 were included in PD.

A problem with DSM-5 criteria is the overlap between PD and AAN. Since the present database lacked data on “significant weight loss”, which characterizes AAN, patients who only purged and fulfilled the psychological criteria could be diagnosed with either PD or AAN (most PD patients fulfilled the psychological criteria for AAN, however these do not apply to PD). This meant that it was possible to determine whether patients fulfilled PD criteria, but not whether they also fulfilled AAN criteria. It should be borne in mind that PD criteria do not require weight loss and there is no specification of what constitutes significant weight loss (within normal weight) for AAN. Using SPSS syntax to generate diagnoses, the PD and AAN groups exchanged patients depending on which diagnosis was defined first. By changing the order of definition, the PD group decreased from 184 to 4 patients. In the present study, the syntax solution that generated the most PD cases was used, since this definition of PD was true to DSM-5 whereas the AAN definition was incomplete.

### Definition of remission

Within the group lacking a formal DSM-IV diagnosis according to the SEDI interview at follow-up, several patients had some remaining symptoms and fulfilled criteria for ‘EDNOS Other’. Twenty one percent of all follow-ups, or about one third of the patients with no DSM-IV ED-diagnosis, still had some ED symptoms. Patients were judged to be in remission if they neither fulfilled criteria for a DSM-IV ED nor ‘EDNOS Other’.

### Analyses

Diagnostic groups were compared on the dependent variables using univariate ANOVAs. Alpha levels were set to *p* < 0.01 for both omnibus and *post hoc* tests, and *η*_*p*_^2^ (≥0.01 = small effect, ≥0.06 = medium effect, and ≥0.14 = large effect) and Cohen’s *d* (≥0.2 = small effect, ≥0.5 = medium effect, and ≥0.8 = large effect) were used to assess effect sizes [45]. To check for differences between groups, *post hoc* Scheffé tests were used. To avoid inclusion of statistically significant results with no practical or clinical significance, pairwise *post hoc* contrasts required both a *p* < 0.01 and a Cohen’s *d* ≥0.50. Welch’s ANOVA and Games-Howell’s *post hoc* were used with unequal and small group sizes in combination with unequal variances. ANOVA on residual gain scores was used to analyze differences in change from initial assessment to follow-up. χ^2^ and Fisher’s exact test were used on categorical data, and alpha was set to *p* < 0.01. *Post hoc* analyses of larger contingency tables were conducted through multiple pairwise tests and inspection of standardized residuals; alpha was set to *p* < 0.001. Cramer’s V/Phi coefficients were used for measuring strength of associations with Rea and Parkers’ [46] magnitude criteria: <0.10 = negligible, 0.10 - < .20 = weak, 0.20 - < 0.40 = moderate, 0.40 - < 0.60 relatively strong, 0.60 - < 0.80 = strong, ≥ 0.80 = very strong.

BED was omitted from analyses of purging behavior and inappropriate compensatory behavior since these behaviors should not be present in the diagnosis. For the same reasons RAN was omitted from analyses of OBE and purging behavior and PD from the analyses of OBE. For all statistical analyses, SPSS v. 19 was used.

### Outliers

Inspection of data found outliers in EDE-Q questions concerning frequency of symptom behavior, such as vomiting, use of laxatives, diuretics, and excessive exercise. To deal with problematic outliers extreme values were first adjusted to a pre-defined reasonable maximum value. All values were subsequently z-transformed for each diagnostic group, and values/groups of values more than 1 *S*.*D*. from the next highest score were deleted.

## Results

Since the present study was carried out prior to DSM-5 publication, we initially included the previously proposed, but later removed, subcategory of subthreshold BED. The decision to remove this subgroup from DSM-5 appears reasonable given our data since we found only 11 such cases (i.e. only 0.5% of the sample); these patients are not considered further in the analysis. Table 1 shows the distribution of diagnoses, BMI, and age when applying DSM-5 criteria. PD was found in 8.2% of patients, which was 32% of the OSFED group. In the OSFED group PD, AAN and Sub-BED together made up 88% (there were no SubBN patients), while the remaining patients were diagnosed with UFED.

**Table 1:** **Distribution of DSM**-**5 diagnoses**, **BMI**, **and age according to diagnosis**

			**BMI**	**Age**	**12**-**month**
**DSM**-**5 diagnosis**	**N**	%	***M*** (***SD***)	***M*** (***SD***)	**N**
RAN	364	16.3	16.1 (1.6)	23.2 (6.6)	102
ANB/P	295	13.2	16.8 (1.4)	24.3 (7.1)	67
BN	874	39.1	23.6 (4.3)	26.4 (8.0)	219
BED	124	5.6	31.4 (7.7)	30.2 (10.2)	22
AAN	310	13.9	22.7 (4.5)	26.0 (7.0)	69
Sub BED	11	0.5	30.2 (7.6)	34.4 (13.2)	-
PD	184	8.2	21.9 (3.5)	25.4 (7.9)	39
UFED	71	3.2	19.9 (6.7)	26.1 (9.1)	12

*RAN* Restrictive Anorexia nervosa, *ANB/P* Anorexia Nervosa Bingeing/Purging type, *BN* Bulimia Nervosa, *BED* Binge Eating Disorder, *AAN* Atypical Anorexia Nervosa, *Sub BED* Subthreshold Binge Eating Disorder, *PD* Purging Disorder, *UFED* Unspecified Feeding and Eating Disorder, *BMI* Body Mass Index.

### Are DSM-5 diagnoses statistically and clinically distinct at presentation?

#### ED variables

To be significant, all pairwise *post hoc* contrasts required both *p* < 0.01 and Cohen’s *d* ≥0.50. For categorical data, *p* < 0.001 was required. Significant ANOVAs were obtained for both the EDE-Q and the CIA with *η*_*p*_^2^ showing a large and a medium effect, respectively (Table 2). Post-hoc tests showed that the UFED group had less severe ED pathology and impairment compared to all other groups. On EDE-Q global scale, ANB/P, BN and PD reported greater severity of ED psychopathology and differed significantly from RAN, with medium effects. BN also differed from AAN and BED. Besides the lesser impairment reported in the UFED group, ANB/P patients experienced more impairment on the CIA than AAN and PD patients.

**Table 2:** **Means**, **standard deviations**, **and significant differences** (**ANOVA**) **on measures of ED and general psychopathology according to DSM**-**5 diagnoses**^**a**^

**Dependent variable**	**RAN**	**ANB**/**P**	**BN**	**BED**	**AAN**	**PD**	**UFED**		
	**Mean** (**SD**)	**Mean** (**SD**)	**Mean** (**SD**)	**Mean** (**SD**)	**Mean** (**SD**)	**Mean** (**SD**)	**Mean** (**SD**)	**F**	**η**_**p**_^**2**^
EDE-Q Global^1^	3.38 (1.36)	4.18 (1.18)	4.20 (0.95)	3.67 (1.08)	3.64 (1.16)	4.09 (0.97)	1.72 (1.22)	77.64***	.174
CIA^2^	29.07 (11.4)	33.61 (9.9)	30.51 (9.3)	29.82 (9.2)	26.39 (10.5)	28.41 (9.5)	19.19 (11.6)	24.27***	.070
CPRS ^3^	1.14 (.51)	1.34 (0.52)	1.14 (.47)	1.14 (.39)	1.08 (.49)	1.14 (.49)	0.76 (.50)	16.22***	.042
SASB Affiliation^4^	-10.98 (37.2)	-28.3 (31.4)	-16.9 (33.0)	-21.4 (33.4)	-11.1 (35.2)	-16.0 (34.5)	17.3 (41.6)	20.48***	.053
SASB Self-emancipation	30.4 (15.6)	31.2 (17.0)	32.0 (15.4)	35.1 (17.1)	31.2 (15.3)	30.6 (14.2)	38.6 (18.6)	3.95***	.011
SASB Self-control^5^	62.1 (18.3)	59.8 (17.2)	54.8 (18.5)	49.5 (20.8)	57.0 (17.9)	53.7 (18.9)	52.5 (19.7)	12.72***	.033

*RAN* Restrictive Anorexia nervosa, *ANB/P* Anorexia Nervosa Bingeing/Purging type, *BN* Bulimia Nervosa, *BED* Binge Eating Disorder, *AAN* Atypical Anorexia Nervosa, *PD* Purging Disorder, *UFED* Unspecified Feeding and Eating Disorder, *EDE-Q* Global Eating Disorder Examination Questionnaire Global score, *CIA* Clinical Impairment Assessment, *CPRS* Comprehensive Psychiatric Rating Scale, *SASB* Structural Analysis of Social Behavior.

^a^For a significant post hoc difference both *p* < 0.01 and Cohen’s *d* ≥0.50 were required; *** *p* < 0.001.

^1^UFED < all; RAN < ANB/P, BN, PD; BN > BED, AAN.

^2^UFED < all; ANB/P > AAN, PD.

^3^UFED < all; ANB/P > AAN.

^4^UFED < all; ANB/P < RAN, AAN.

^5^ BED < RAN, ANB/P.

##### *Objective Binge Episodes* (*OBE*)^c^

Table 3 shows frequency and percent of patients reporting OBE. Based on the EDE-Q, 56% of all patients reported OBE. In BN and BED, 74 and 84% respectively reported OBE compared to about half of the patients with ANB/P and AAN. Among RAN and PD, 25 and 38% respectively reported OBE even though this should not be present regularly in these diagnoses. χ^2^ indicated an overall significant difference (*p* < 0.001) between groups and pairwise tests showed that OBE was more frequent among BN and BED compared to the other groups, with effects ranging from weak to moderate for BN: i.e. phi = 0.18 (BN vs. ANB/P) to 0.25 (BN vs. AAN); and from moderate to relatively strong for BED i.e. phi = 0.26 (BED vs. AN/BP) to 0.50 (BED vs. UFED). More patients with ANB/P reported OBE compared to UFED (phi = 0.17). Welch’s ANOVA showed a significant overall difference (*p* < 0.001) in mean frequency during the last month (Table 4), and Games-Howell showed that ANB/P, BN, and BED reported more OBE than AAN and UFED (excluding RAN and PD), with medium/large Cohen’s *d* for all comparisons, except for ANB/P vs. AAN which almost reached a medium effect (*d* = 0.49).

**Table 3:** **Number**, **percent and overall χ**^**2 **^**for eating disorder behaviors on the EDE**-**Q according to DSM**-**5 diagnoses**^**a**^

	**RAN**	**ANB**/**P**	**BN**	**BED**	**AAN**	**PD**	**UFED**	**Total**	**χ**^**2**^ (**df**, **N**)
**EDE**-**Q symptom**	**N (%)**	**N (%)**	**N (%)**	**N (%)**	**N (%)**	**N (%)**	**N (%)**	**N (%)**	
OBE^1^	93 (25)	163 (55)	646 (74)	103 (83)	146 (47)	70 (38)	24 (34)	1245 (56)	133,94*** (4, 1674)
SBE^2^	216 (59)	205 (70)	524 (60)	44 (36)	179 (58)	122 (66)	16 (23)	1306 (59)	85,27*** (6, 2221)
Vomiting^3^	27 (7)	230 (78)	621 (71)	8 (6)	67 (22)	150 (82)	21 (30)	1124 (51)	340,79*** (4, 1734)
Laxatives^4^	8 (2)	60 (20)	138 (16)	2 (2)	5 (2)	33 (18)	1 (1)	247 (11)	64,52*** (4, 1734)
Diuretics^5^	4 (1)	29 (10)	46 (5)	1 (1)	9 (3)	12 (7)	1 (1)	102 (5)	17,11** (4, 1734)
Exercise^6^	155 (43)	150 (51)	473 (54)	13 (11)	153 (49)	102 (55)	13 (18)	1059 (48)	45,35*** (5, 2098)
Extreme fasting^7^	210 (58)	227 (77)	576 (66)	45 (36)	180 (58)	128 (70)	13 (18)	1379 (62)	99,75*** (5, 2097)

*RAN* Restrictive Anorexia nervosa, *ANB/P* Anorexia Nervosa Bingeing/Purging type, *BN* Bulimia Nervosa, *BED* Binge Eating Disorder, *AAN* Atypical Anorexia Nervosa, *PD* Purging Disorder, *UFED* Unspecified Feeding and Eating Disorder, *OBE* Objective Binge Episodes, *SBE* Subjective Binge Episodes.

^a^BED was omitted from all analyses except for OBE and SBE since purging behavior and inappropriate compensatory behavior should not be present in the diagnosis. For the same reason RAN was omitted from the analyses of OBE and purging behavior, and PD from the analyses of OBE. *** *p* < 0.001; ** *p* < 0.01.

^1^BN, BED > all; ANB/P > UFED.

^2^BED, UFED < all.

^3^AAN, UFED < all.

^4^ANB/P, BN, PD > all.

^5^ANB/P > AAN.

^6^UFED < all; RAN < BN.

^7^ANB/P > RAN, BN, AAN; UFED < all.

**Table 4:** **Mean frequency**, **standard deviation and significant differences of eating disorder psychopathology during last four weeks according to DSM**-**5 diagnoses**^**a**,**b**^

**Dependent variable**	**RAN**	**ANB**/**P**	**BN**	**BED**	**AAN**	**PD**	**UFED**	***F***	**η**_**p**_^**2c**^
OBE^1^	7.8 (7.9)	14.0 (15.5)	12.7 (9.9)	14.6 (7.9)	8.1 (7.1)	8.8 (7.2)	5.3 (3.4)	11.06***	.040
SBE^2^	11.3 (8.9)	12.0 (8.0)	10.9 (8.0)	11.3 (8.8)	9.8 (7.2)	9.8 (7.6)	6.1 (3.4)	6.27***	.012
Vomiting^3^	3.0 (2.6)	23.8 (24.9)	20.0 (21.2)	3.3 (1.6)	15.1 (19.7)	14.0 (13.2)	12.2 (10.1)	9.55***	.023
Laxatives^4^	2.0 (1.1)	9.6 (8.3)	11.5 (10.2)	1.0 (0)	4.8 (3.4)	8.3 (8.6)	4.0		
Diuretics^5^	2.5 (2.1)	11.2 (9.8)	12.7 (9.5)	2.0	4.6 (3.3)	10.4 (4.4)	---		
Exercise	15.9 (9.0)	15.7 (7.8)	13.2 (7.0)	6.7 (3.6)	15.5 (8.3)	14.8 (7.9)	14.3 (9.5)	4.74***	.023
Extreme fasting ^d; 6^	1.79 (2.1)	2.69 (2.2)	1.76 (1.9)	0.69 (1.2)	1.68 (2.0)	2.10 (2.1)	0.38 (1.1)	35.30***	.046

*RAN* Restrictive Anorexia nervosa, *ANB/P* Anorexia Nervosa Bingeing/Purging type, *BN* Bulimia Nervosa, *BED* Binge Eating Disorder, *AAN* Atypical Anorexia Nervosa, *PD* Purging Disorder, *UFED* Unspecified Feeding and Eating Disorder, *OBE* Objective Binge Episodes, *SBE* Subjective Binge Episodes.

^a^For a significant post hoc difference both *p* < 0.01 and a Cohen’s *d* ≥0.50 were required.

^b^BED was omitted from all analyses except for OBE and SBE since purging behavior and inappropriate compensatory behavior should not be present in the diagnosis. For the same reason RAN was omitted from the analyses of OBE and purging behavior and PD from the analyses of OBE. Also, groups with n < 10 were omitted.

^c^η_p_^2^ has been calculated through a regular ANOVA, since it was not a possible option in the Welch analysis.

^d^Answers for ‘extreme fasting’ is 0 = no days, 1 = 1-2 days, 2 = 3-6 days, 3 = 7 days, 4 = 8-10 days, 5 = 12-13 days, 6 = every day.

***p < .001;

^1^ANB/P, BN, BED > AAN, UFED.

^2^UFED < RAN, ANB/P, BN.

^3^UFED < ANB/P.

^4^ANB/P, BN and PD included in the analysis. AAN and UFED not included due to small *n*.

^5^ANB/P, BN and PD included in the analysis. AAN and UFED not included due to small *n*.

^6^UFED < all; ANB/P > RAN, BN, AAN.

##### Subjective Binge Episodes (SBE)

Based on the EDE-Q, 59% reported SBE. Among RAN, ANB/P, BN, AAN and PD between 58% and 70% of the patients reported SBE, whereas the corresponding percentages in BED and UFED were 36% and 23%, respectively (Table 3). χ^2^ indicated significant differences between groups, and pairwise tests suggested that SBE was less frequent among BED and UFED compared to the other diagnoses showing moderate effects (phi = 0.20 to 0.39), except for BED vs. BN, which was weak (phi = 0.16). Welch’s ANOVA showed a significant overall difference (*p* < 0.001) in mean frequency during the last month (Table 4), and Games-Howell showed that UFED reported fewer SBE than RAN, ANB/P and BN with medium/large Cohen’s *d* for all comparisons.

##### Compensatory behaviors

Table 3 shows number, percent and χ^2^ of patients reporting compensatory behaviors on EDE-Q^d^. Table 4 shows mean frequencies/last 28 days and significant differences for the same behaviors. χ^2^ suggested significant differences on all compensatory behaviors.

*Regular vomiting* was reported by 82% of the PD group compared to 78% and 71% in the ANB/P and BN group, respectively. In the UFED and AAN groups, the percentages were 30% and 22%, respectively, which was significantly less compared to the other groups (excluding RAN and BED). Effects were relatively strong for AAN (phi = 0.44 to 0.58) and moderate to relatively strong for UFED (phi = 0.23 to 0.50). Welch analysis on mean frequencies/28 days was significant, and Games-Howell post-hoc showed that PD reported less vomiting compared to ANB/P and BN. However, Cohen’s *d* showed only small effects for both comparisons. Also, UFED reported less vomiting compared to ANB/P with a medium effect.

*Laxative use* was reported significantly more often in ANB/P, BN, and PD than in other groups, with moderate effects for ANB/P and PD (phi = 0.20 to 0.30) and weak to moderate effects for BN (phi = 0.11 to 0.20). There were no significant differences between these groups in mean frequency/28 days (omitting AAN and UFED due to small *n*’s).

Use of *diuretics* was relatively uncommon, but was reported by 5% to 10% of patients with BN, PD and ANB/P. In AAN 3% reported the use of diuretics which was significantly less compared to ANB/P, however the effect was weak (phi = 0.14). Only ANB/P, BN and PD were included in the ANOVA of mean frequency/28 days, which suggested no significant difference.

*Strenuous exercise* for the purpose of weight control was reported to the same extent in PD, BN, ANB/P, and AAN, and to a much lesser degree in UFED (phi = 0.19 to 0.33). Strenuous exercise was also reported by fewer patients with RAN compared to BN, although with a weak effect (phi = 0.12). ANOVA/Welch was significant overall for mean frequency/28 days, and Games-Howell suggested a significantly higher frequency in ANB/P than in BN (*p* < 0.01), but Cohen’s *d* showed only a weak effect.

*Extreme fasting*/*dieting* was most commonly reported in the ANB/P group followed by PD and BN. In the AAN and RAN groups close to 60% reported this behavior. It was significantly more often reported in ANB/P compared to RAN, BN, and AAN (phi = 0.20/0.10/0.20 respectively), and significantly less often in UFED compared to the other groups, with moderate effects for RAN, BN and AAN (phi = 0.26 to 0.31) and relatively strong effects for ANB/P and PD (phi = 0.49 and 0.46 respectively (excluding BED)). Additionally, UFED reported a significantly lower frequency/28 days compared to other groups. ANB/P frequency was significantly higher than RAN, BN and AAN, but Cohen’s *d* showed small effects.

#### Psychiatric and personality variables

Means for the CPRS and SASB ‘Affiliation’, ‘Self-emancipation’, and ‘Self-control’ are shown in Table 2. On the CPRS, the UFED group reported fewer problems than the other groups, and ANB/P reported more problems compared to AAN. Likewise, on SASB ‘Affiliation’, UFED reported significantly better self-image compared to the other groups, and ANB/P reported more negative self-image compared to AAN and RAN. BED patients scored significantly lower on SASB ‘Self-control’ compared to RAN and ANB/P patients. All *η*_*p*_^2^ on the omnibus tests showed small effects.

### *Psychiatric co*-*morbidity*

Table 5 presents numbers, percentages and overall significance (χ^2^) for mood, anxiety, and alcohol/substance abuse or dependency disorders for each diagnostic group.

**Table 5:** **Frequency**, **percent and overall significance** (**χ2**) **for Axis I co**-**morbidity according to DSM**-**5 diagnoses**

	**RAN**	**ANB**/**P**	**BN**	**BED**	**AAN**	**PD**	**UFED**	**Total**	
	**N** = **364**	**N** = **295**	**N** = **874**	**N** = **124**	**N** = **310**	**N** = **184**	**N** = **71**	**N** = **2222**	**χ**^**2**^(**df**, **N**)
Axis I disorders	N (%)	N (%)	N (%)	N (%)	N (%)	N (%)	N (%)	N (%)	
Mood disorders^1^	104 (29)	138 (47)	417 (48)	71 (57)	116 (37)	66 (36)	11 (15)	923 (42)	79,31***(6, 2222)
Anxiety disorders	47 (13)	53 (18)	143 (16)	21 (17)	51 (16)	35 (19)	6 (8)	365 (16)	7,90 (6, 2222)
Substance use disorders^2^	13 (4)	31 (11)	120 (14)	19 (15)	32 (10)	21 (11)	6 (8)	242 (11)	30,50***(6, 2222)

*RAN* Restrictive Anorexia nervosa, *ANB/P* Anorexia Nervosa Bingeing/Purging type, *BN* Bulimia Nervosa, *BED* Binge Eating Disorder, *AAN* Atypical Anorexia Nervosa, *PD* Purging Disorder, *UFED* Unspecified Feeding and Eating Disorder.

***p < 0.001.

^1^UFED < all except RAN; BED > RAN, AAN, PD; RAN < ANB/P, BN.

^2^RAN < all except UFED.

### Mood disorders

UFED had a significantly lower rate of mood disorders compared to all other groups except RAN, ranging from weak (BN, AAN) to moderate/relatively strong (PD, ANB/P, BED) effects (phi = 0.17 to 0.41). There were medium effects between BED and RAN (*p* < 0.001; phi = 0.26), and BED and PD (*p* < 0.001; phi = 0.21), and a weak effect for BED vs. AAN (*p* < 0.001; phi = 0.18). RAN also differed significantly from BN and ANB/P, but with weak effects (*p* < 0.001; phi = 0.18 and 0.19 respectively).

### Anxiety disorders

19% of the PD group had an anxiety disorder compared to only 8% of the UFED group. For other EDs, percentages varied between 13 and 18. However, the overall test did not reach significance.

### Substance use disorders

RAN had a significantly lower rate of substance use disorders compared to all other groups (*p* < 0.001), except UFED. However, all effects were weak (phi = 0.13 - 0.15), apart from the RAN-BED difference, which was moderate (phi = 0.21).

#### Do DSM-5 diagnoses predict outcome after one year?

ANOVA on residual gain scores for the 12-month follow-up assessments did not show any significant differences between diagnoses on either the ED (EDE-Q and CIA) or the psychiatric/personality (CPRS and SASB) variables.

#### Remission rates and diagnostic stability

Table 6 presents remission rates and diagnostic cross-over/stability at 12-months. UFED had the highest remission rate (but a very low N) and BED and AN the lowest, but there were no significant differences in remission rates between diagnoses. Among PD patients, 44% were in remission at 12-months and 41% had crossed over to other diagnoses, which was very similar to BN. On average, 39% (range 17 to 55%) of all patients fulfilled criteria for an ED diagnosis other than their initial one at follow-up. If, however, migration to UFED, which was the only diagnosis that had increased at follow-up, and AAN (which only decreased slightly) was not included, migration to other diagnoses was on average 14% (i.e. from 4% for RAN to 25% for ANB/P).

**Table 6:** **Diagnostic stability**/**cross**-**over and remission rates at 12**-**month follow**-**up**

	**DSM**-**5 diagnoses at 12**-**month follow**-**up**								
**DSM**-**5 diagnoses at start**	**RAN**	**ANB**/**P**	**BN**	**BED**	**AAN**	**PD**	**UFED**	**No diagnosis**/ **remission**	**Total**
RAN	29 (28%)	2 (2%)	2 (%)	0	17 (17%)	0	18 (18%)	34 (33%)	102 (19%)
ANB/P	6 (9%)	13 (19%)	6 (9%)	0	7 (10%)	5 (7%)	11 (16%)	19 (28%)	67 (13%)
BN	1(0.5%)	3 (1%)	42 (19%)	2 (1%)	20 (9%)	5 (2%)	54 (25%)	92 (42%)	219 (41%)
BED	0	0	4 (18%)	3 (14%)	2 (9%)	0	6 (27%)	7 (32%)	22 (4%)
AAN	0	1 (1%)	6 (9%)	0	13 (19%)	4 (6%)	14 (20%)	31 (45%)	69 (13%)
PD	1 (3%)	1(3%)	2 (5%)	0	4 (10%)	6 (15%)	8 (21%)	17 (44%)	39 (7%)
UFED	1 (8%)	0	1 (8%)	0	0	0	4 (33%)	6 (50%)	12 (2%)
Total	38 (7%)	20 (4%)	63 (12%)	5 (1%)	63 (12%)	20 (4%)	115 (22%)	206 (39%)	530

*RAN* Restrictive Anorexia nervosa, *ANB/P* Anorexia Nervosa Bingeing/Purging type, *BN* Bulimia Nervosa, *BED* Binge Eating Disorder, *AAN* Atypical Anorexia Nervosa, *PD* Purging Disorder, *UFED* Unspecified Feeding and Eating Disorder.

## Discussion

The present study examined the clinical characteristics and distinctiveness of the new DSM-5 ED diagnoses in general and PD in particular. To our knowledge this is the most comprehensive study to date, including PD and other OSFED subtypes, as well as UFED, assessed on general psychopathology, ED variables, psychiatric co-morbidity and outcome.

Strengths of the study include its large-scale naturalistic setting and the inclusion of the full range of EDs, ensuring good generalizability to clinical settings. However, some limitations should be noted. First, it is possible that a treatment-seeking sample may differ from a community sample in severity and psychiatric co-morbidity. Second, there was considerable attrition at follow-up. Statistical analyses, however, suggested that this did not impact on results. Third, both clinical interview and questionnaire data were used to match DSM-5 criteria as closely as possible. However, due to lack of data the match was not perfect for duration and frequency, and there were no data on the unspecified criterion of “significant weight loss” necessary for AAN. Finally, it is possible that the more lenient DSM-5 criteria for AN and BN used here might have led to inclusion of patients with milder symptoms, potentially decreasing levels of psychopathology and affecting the comparability of the present study and other PD-studies using DSM-IV criteria. However, means for all psychopathology variables for AN and BN using DSM-IV definitions were only marginally elevated compared to those for the DSM-5 definitions.

A main finding was that there were no statistically significant or clinically meaningful differences between PD and BN, and the only difference between PD and ANB/P was on the CIA. Also, PD did not differ from any other diagnosis concerning change in psychopathology or remission rates from start to follow-up, which supports studies finding no differences in treatment outcome between PD, AN and BN [20,21]. SBE has been suggested to be of special importance in defining PD [47], but in the present sample it was common in several diagnoses and not distinctive for PD. Taken together, the present results suggest no grounds for considering PD as a distinct ED.

On the contrary, PD, AN and BN seem to be more similar to each other than distinct. Patients with PD, ANB/P and BN scored higher on the EDE-Q compared to RAN, which is in line with Tasca *et al*. [20] and studies finding fewer problems in RAN than ANB/P and BN despite a very serious medical condition [48]. In a meta-analysis, Keel [17] found no meaningful difference between PD and BN concerning ED severity. Keel suggested that a higher ‘eating concern’ in BN could distinguish the two disorders; however, the argument risks becoming circular. If one ED includes binge eating, which is associated with considerable distress, and the other does not, then higher eating concern in the former is to be expected. PD did not differ significantly from BED on measures of psychopathology, but more patients with PD reported SBE. The only significant difference between PD and AAN was more purging behavior in PD. This could partly be explained by the fact that ‘purging only’ patients who could have been categorized as AAN were included in the PD group. The conceptual overlap between conditions due to the absence of operationalization of “significant weight loss” in DSM-5 makes this question difficult to evaluate.

OBE measured through the EDE-Q was reported to a relatively high degree among patients with RAN and PD even though the behavior should not be present “regularly” in these diagnoses. Moreover, not all patients with BN or BED reported the behavior through self-report. The fact that self-reports and interview data might differ in various directions has been noted earlier [49-52] and especially the tendency to under-diagnose BN using self-report questionnaires [50]. Moreover, the SEDI has only been validated against the EDE interview [27], and no study has so far investigated the concordance between SEDI and EDE-Q. In the present study, self-reported OBE (EDE-Q), was counted as present if the patient reported the behavior at least once over a period of 28 days. This might not count as “regularly” according to the DSM criterion, which may help to explain the presence of OBE in RAN and PD. However, it was the interview data (SEDI) that formed the basis for the DSM-5 diagnoses, and EDE-Q frequency data were only used to establish the lower DSM-5 criteria in cases where patients did not already meet DSM-IV criteria.

Significant differences between ED diagnostic categories on important variables that are not criteria-related (e.g. ED psychopathology, distress, co-morbidity etc.) would support the distinctiveness of these categories [18,19]. However, when such differences are lacking and the distinctiveness of the categories is only supported by differences in variables that are used to define the categories, evidence for the scientific validity and clinical utility of these categories is weakened. Our failure to find differences on variables that are not criteria-related undermines the case for DSM-based diagnostic distinctions and supports a more transdiagnostic and synthetic view of ED. Nevertheless such differences may remain to be uncovered by future research. It will therefore be important to examine ED categories in regard to pivotal factors such as prognosis and outcome.

### Psychiatric co-morbidity

UFED had significantly fewer mood disorders compared to the other groups, with moderately/relatively strong differences compared to PD, ANB/P and BED. PD had a lower rate of mood disorders than BED (just reaching the criteria for a moderate effect). In contrast to Keel *et al*. [18,21] we did not find PD to differ meaningfully from BN on any Axis I disorders. Keel and co-workers’ suggestion that PD patients may be protected from developing considerable problems with OBEs because they have fewer problems with affect regulation, was therefore not supported by the present data. Except for fewer mood disorders in UFED, the only differences showing at least moderate effects were a higher rate of mood disorders in BED compared to RAN and PD, and a higher rate of substance abuse disorders compared to RAN. These latter differences are well in line with studies linking binge eating to substance abuse, and problems with impulse control and affect regulation [53-55]. In sum, few meaningful differences were found concerning psychiatric co-morbidity between the ED groups.

### Remission and diagnostic stability

About one third of patients with AN or BED no longer had an ED at follow-up compared to almost 60% of UFED. This striking difference was not significant though, probably due to a very small UFED group. In accordance with other studies, we found no difference in remission rates between PD and other ED groups. Comparable to BN, about 40% of the PD group was in remission at follow-up, which is similar to other studies [13]. At follow-up, 41% of patients with PD met criteria for another ED, a figure comparable to BN. The large migration, especially to UFED but also to AAN, may reflect a partial remission of illness rather than a “true” change of diagnosis.

The observed diagnostic instability is troublesome for DSM, and suggests that many symptoms vary over time and are not merely an effect of recovery [10-12]. Many patients who are in partial remission or improved will fulfill criteria for a new diagnosis (i.e. EDNOS/OSFED/UFED) in both DSM-IV and DSM-5. According to Agras and co-workers [15], EDNOS may be a way-station between full syndromes and non-ED. It is probable that many EDs vary in both severity and type of symptoms over time, while causal and maintaining psychopathological processes remain largely the same. A diagnostic system capable of accounting for such symptom fluctuations would not only better capture the general patterns found in diagnostic research, but also be more valuable in clinical practice. One suggestion in this direction is the transdiagnostic model proposed by Fairburn and Bohn [56], which emphasizes the similarities among the diagnoses rather than the often transient differences in symptom presentations. An important challenge facing future revisions of DSM will be to integrate ideas about temporal changes in symptoms. Other possible approaches for defining ED could examine personality characteristics [57] or empirically derived clusters/dimensions [58-60]. Research suggests that dimensional measures of psychopathology generally increase both reliability and validity by 15% to 37%, especially in clinical samples [61]. Such a dimensional approach may better capture symptom fluctuations.

### UFED

The UFED group had considerably less severe problems compared to other groups, which raises the question of where to draw the line between normal and pathological eating behavior. In the absence of a clear definition of what constitutes an ED, this delineation relies on clinical judgment [14]. In the present study, UFED mostly consisted of patients who did not receive a formal DSM-IV ED diagnosis, but still fulfilled inclusion criteria for “EDNOS Other”. One possible interpretation of the milder psychopathology observed in this group could be that somewhere in the interval covered by the UFED the boundary between ED and “normal” eating behavior should be drawn.

### DSM-5 definition of PD and AAN

The DSM-5 definitions of AAN and PD are problematic because of their overlapping descriptions combined with the absence of a definition of “significant weight loss” for AAN. However, nothing is mentioned about weight loss in the description of PD (earlier studies of PD have not determined the absence of significant weight loss, and perhaps they have partly studied AAN patients). As a consequence, it is difficult to determine the presence of PD without having first excluded AAN. Nevertheless, the suggestion that PD might constitute a distinct disorder requires a less ambiguous AAN definition. A definition would have to consider amount of weight, rate or time period of weight loss, and possibly initial and end weight. Keel and Striegel-Moore [24] stated that a candidate DSM-5 syndrome should be reliably differentiated from other similar syndromes, and believe this to be true of PD. However, our results find no convincing evidence that PD is a distinct ED diagnosis.

## Conclusions

There appears to be few meaningful differences between AN binge/purge type, PD and BN, and PD does not appear to constitute a distinct diagnosis. In addition, the DSM-5 distinction between atypical AN and PD requires clarification, and there is a need to stipulate a minimal level of psychopathology for UFED. Given the general instability of ED symptoms and diagnoses, along with their highly problematic operationalization and the limited clinical relevance of DSM-5, it is highly doubtful whether further sub-classification will lead to greater clinical utility. Instead, attention should be focused on delineating what is and is not ED.

## Consent

Written informed consent was obtained from the patient for the publication of research reports and any accompanying images based on the collected information.

## Endnotes

^a^Note that the present study does not concern itself with the so called feeding disorders, i.e. the three diagnoses Pica, Rumination Disorder, and Avoidant/Restrictive Food Intake Disorder which has been moved from the category Disorders Usually First Diagnosed in Infancy, Childhood, or Adolescence in DSM-IV to the Feeding and Eating Disorders category in DSM-5. The reason for this is that the study uses a database that is developed for DSM-IV EDs, and thus does not contain the data necessary for studying the feeding disorders. Also, we did not have data for Night Eating Syndrome (NES).

^b^For ‘self-emancipation’ the difference did not reach a small effect, and for ‘self-control’ the effect was small (η_p_^2^ = .015).

^c^OBE is a diagnostic criterion for BN and BED and should not be present (regularly) for the diagnoses RAN and PD. There can however be differences between self-report questionnaires and clinical interviews, the latter being the basis for those diagnoses.

^d^Purging behavior is a diagnostic criterion and should not be present (regularly) for a diagnosis of RAN or BED, which explains the low frequencies of these behaviors in those groups. Also, other inappropriate compensatory behaviors such as excessive exercise and extreme dieting should not be (regularly) present in BED. There can however be differences between self-report questionnaires and clinical interviews, the latter being the basis for those diagnoses.

## Abbreviations

AAN: Atypical anorexia nervosa; AN: Anorexia nervosa; ANB/P: Anorexia nervosa bingeing/purging type; BED: Binge eating disorder; BMI: Body mass index; BN: Bulimia nervosa; CIA: Clinical impairment assessment; CPRS-S-A: Comprehensive psychiatric rating scale - self-rating scale for affective syndromes; EDE-Q: Eating disorder examination questionnaire; OSFED: Other specified feeding and eating disorders; OBE: Objective binge episodes; UFED: Unspecified feeding and eating disorder; PD: Purging disorder; RAN: Restrictive anorexia nervosa; SASB: Structural analysis of social behavior; SBE: Subjective binge episodes; SCID-I: Structured clinical interview for DSM-IV Axis I disorders; SEDI: Structured eating disorder interview.

## Competing interests

One author (Norring, C.) is a member of the ICD-11 revision workgroup. No other conflicts of interest, financial or other, exist.

## Authors’ contributions

All authors planned and designed the study, and discussed and analyzed the results. KE conducted the literature searches, the main statistical analyses, and wrote the first draft of the manuscript. AB, CN, and DC assisted in manuscript revisions and all authors have contributed to and have approved the final manuscript. We had full access to all of the data in the study and take responsibility for the integrity of the data and the accuracy of the data analysis. All authors read and approved the final manuscript.
